# Experimental Infection and Response to Rechallenge of Alpacas with Middle East Respiratory Syndrome Coronavirus

**DOI:** 10.3201/eid2206.160007

**Published:** 2016-06

**Authors:** Gary Crameri, Peter A. Durr, Reuben Klein, Adam Foord, Meng Yu, Sarah Riddell, Jessica Haining, Dayna Johnson, Maged G. Hemida, Jennifer Barr, Malik Peiris, Deborah Middleton, Lin-Fa Wang

**Affiliations:** CSIRO Australian Animal Health Laboratory, Geelong, Victoria, Australia (G. Crameri, P.A. Durr, R. Klein, A. Foord, M. Yu, S. Riddell, J. Haining, D. Johnson, J. Barr, D. Middleton);; Kafrelsheikh University, Kafr Elsheikh, Egypt (M.G. Hemida);; King Faisal University, Hofuf, Saudi Arabia (M.G. Hemida);; University of Hong Kong, Hong Kong, China (M. Peiris);; Duke–National University of Singapore Medical School, Singapore (L.-F. Wang)

**Keywords:** coronavirus, Middle East respiratory syndrome, MERS-CoV, severe acute respiratory syndrome, SARS, camel, alpaca, experimental animal model, infection, rechallenge, shedding, vaccine, viruses, zoonoses

## Abstract

We conducted a challenge/rechallenge trial in which 3 alpacas were infected with Middle East respiratory syndrome coronavirus. The alpacas shed virus at challenge but were refractory to further shedding at rechallenge on day 21. The trial indicates that alpacas may be suitable models for infection and shedding dynamics of this virus.

Middle East respiratory syndrome coronavirus (MERS-CoV) was first reported in September 2012 (*1*); since then, >1,600 confirmed cases have been reported to the World Health Organization (http://www.who.int/csr/don/29-february-2016-mers-saudi-arabia/en). The role of domestic animals as an intermediate host for humans was initially suggested by case histories of infected patients who had visited farms or tended sick animals shortly before onset of infection (*2*). This suggestion was given credence by a study of camel serum samples that showed high levels of neutralizing antibodies in disparate camel populations (*3*); the findings were subsequently confirmed by virus detection and sequencing (*4*).

Infection trials in camels have been limited (*5**,**6*), mainly because of difficulties in housing and handling the animals in a high-containment facility, which is necessary because the virus has a Biosafety Level 3 classification (*7*). However, the alpaca, a close relative within the *Camelidae* family, may provide a temperamentally suitable and valuable animal model for MERS-CoV infection, particularly for developing and testing vaccine candidates for camels. We sought to assess whether alpacas could be infected by means of a natural (oronasal) route, to determine whether viral shedding occurred after reinfection, and to evaluate the development of serologic markers of protection.

## The Study

We obtained 3 adult female alpacas (*Vicugna pacos*) from a commercial supplier in Victoria, Australia, and housed them in the Biosafety Level 3 containment facility at the CSIRO Australian Animal Health Laboratory. Before experiments, the alpacas were allowed to acclimatize for 6 days; during this time, intrauterine temperature data loggers were implanted according to a previously published procedure (*8*). We found no previous MERS-CoV challenge trial reported in alpacas, so we chose a preliminary dose and rechallenge time on the basis of our experience with other virus infection trials for other emerging infectious diseases (*8*).

We used a camel isolate of MERS-CoV (Dromedary_MERS-CoV_Al-Hasa_KFU-HKU13/2013; GenBank accession nos. KJ650295–KJ650297) for infection; the isolate was prepared in Vero cells as described (*9*). The 3 alpacas were exposed oronasally to a 10^6^ 50% tissue culture infective dose of MERS-CoV in 5 mL of phosphate-buffered saline. The animals were monitored for 21 days, reexposed to a replicate challenge of MERS-CoV, and observed for 14 more days. Clinical samples of blood (in EDTA for obtaining serum) and swabs (deep and superficial nasal, oral, rectal, and urogenital) were collected immediately before inoculation and thereafter on days 3, 5, 7, 10, 12, 14, 21, 26, 28, 31, 33, and 35. Alpacas were electively euthanized, 1 on day 33 and the others on day 35.

The animals remained clinically healthy except for a reduced condition score that occurred by day 18 in 1 animal (alpaca 2); no signs of upper or lower respiratory tract disease appeared in any animal. Increased temperature was noted in alpaca 2 during days 17–20, but fever (rectal temperature >39°C) was not recorded. Gross abnormalities at postmortem examination were found only in alpaca 2 and comprised extensive adhesions of the caudal sac of compartment 1 of the stomach to the umbilicus; clinical findings in this animal were attributed to this lesion.

RNA extraction and real-time PCR were performed by following specimen-handling procedures established for Hendra virus (*8*) and were used to identify shedding patterns after each challenge. After initial challenge, viral RNA was detected in each animal from oral and deep and superficial nasal swab samples taken on days 3–12 (Table 1).

**Table 1:** Virus shedding in 3 alpacas infected with MERS-Cov, as measured by virus isolation and real-time PCR for each sample day*

Dpi	Cycle threshold value (virus isolation result)†‡												No. positive/no. tested	
	Deep nasal swab sample				Oral swab sample				Superficial nasal swab sample					
	Alpaca 1	Alpaca 2	Alpaca 3		Alpaca 1	Alpaca 2	Alpaca 3		Alpaca 1	Alpaca 2	Alpaca 3		Real-time PCR	Virus isolation
0	U (–)	U (–)	U (–)		U (–)	40.8 (–)	U (–)		U (–)	U (–)	U (–)		0/3	0/3
3	**33.4 (+)**	29.0 (–)	U (–)		**34.2 **(–)	**31.7 **(–)	42.3 (–)		**35.4 **(–)	40.7 (–)	U (–)		2/3	1/3
5	34.9 (–)	**33.5 **(–)	**34.2 **(–)		**32.0 **(–)	**35.4 **(–)	**32.0 (+)**		**35.0 **(–)	**33.0 **(–)	**32.5 **(–)		3/3	1/3
7	**29.4 (+)**	**18.2 **(–)	**31.4 (+)**		**32.7 **(–)	**30.1 (+)**	**28.3 (+)**		**31.9 **(–)	**28.5 (+)**	**38.6 (+)**		3/3	3/3
10	41.0 (–)	**37.5 (+)**	U (–)		41.3 (–)	**38.0 **(–)	**30.5 (+)**		**39.9 **(–)	36.0** (+)**	U (–)		3/3	2/3
12	42.0 (–)	**36.4 **(–)	U (–)		U (–)	U (–)	**37.3 (+)**		42.0 (–)	**39.5 (+)**	U (–)		2/3	2/3
14	U (–)	42.2 (–)	U (–)		43.0 (–)	44.0 (–)	43.0 (–)		U (–)	U (–)	U (–)		0/3	0/3
21	U (–)	U (–)	U (–)		U (–)	U (–)	U (–)		U (–)	U (–)	U (–)		0/3	0/3
24	U (–)	U (–)	U (–)		40.8 (–)	U (–)	U (–)		U (–)	43.2 (–)	U (–)		0/3	0/3
26	U (–)	U (–)	U (–)		U (–)	U (–)	U (–)		U (–)	U (–)	U (–)		0/3	0/3
28	U (–)	U (–)	U (–)		U (–)	U (–)	U (–)		U (–)	U (–)	U (–)		0/3	0/3
31	U (–)	U (–)	U (–)		U (–)	U (–)	U (–)		U (–)	U (–)	U (–)		0/3	0/3
33	U (–)	U (–)	U (–)		U (–)	U (–)	U (–)		U (–)	43.1 (–)	U (–)		0/3	0/3
35§	U (–)	NA	U (–)		U (–)	NA	U (–)		U (–)	NA	U (–)		0/2	0/2

*Bold indicates positive results (cycle threshold <40). Gray shading indicates that >1 animal was positive for the sample collected on that day. Dpi, day postinfection for challenge (initial infection); MERS-CoV, Middle East respiratory syndrome coronavirus; NA, not available; U, undetermined; –, negative; +, positive. †Real-time PCR cycle threshold values are an average of the duplicates, except when 1 result was undetermined; then, only the single numeric value is shown. ‡The starting dilution was 1:10; the threshold for a positive result was a dilution of >1:20.  §Alpaca 2 was euthanized at day 33, leaving only 2 animals in the study at day 35.

Virus isolation was undertaken with Vero cells by using published protocols (*9*) and was successful for all 3 animals from all types of samples. Virus recovery was successful from oral and superficial nasal swab samples through day 12; deep nasal swab samples were positive only through day 10. All urogenital and rectal swab samples were negative by both real-time PCR and virus isolation. After rechallenge, viral RNA was not detected with confidence from any sample (Figure).

**Figure:**
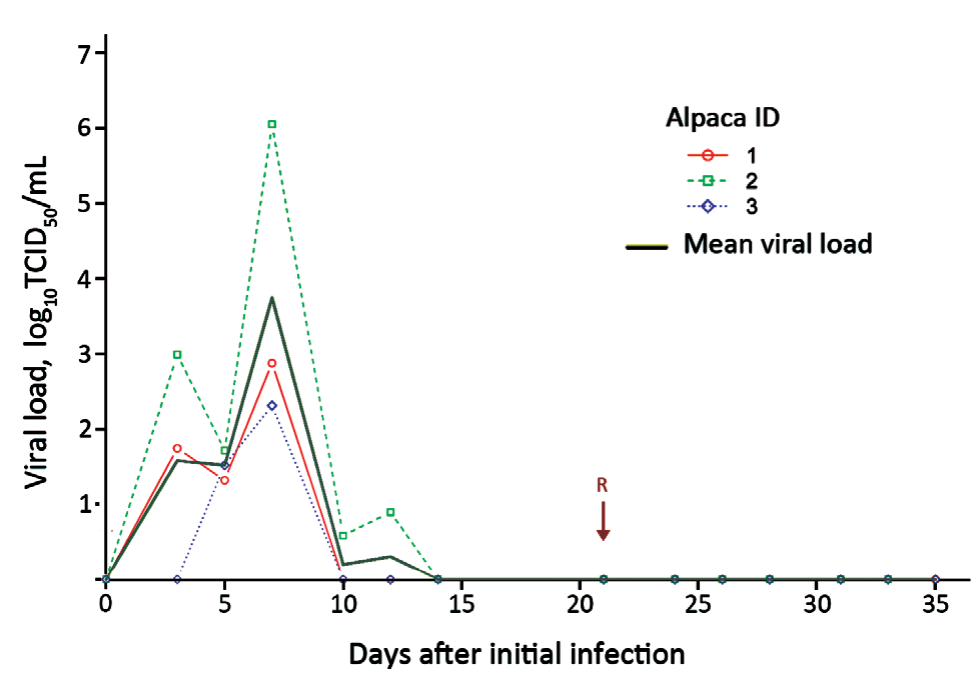
Virus shedding of MERS-CoV from 3 infected alpacas as detected from the deep nasal swab samples by day after initial infection and reinfection. Viral load was estimated from real-time cycle threshold values and a calibration experiment. Arrow indicates day 21, when the animals were reinfected with MERS-CoV. MERS-COV, Middle East respiratory syndrome coronavirus; TCID, tissue culture infective dose.

Serum samples were assessed for immunologic responses by using a virus neutralization test (VNT) and a Luminex bead assay to the nucleocapsid protein. We used in-house assays modeled after those previously developed to assess the serologic status of feral camels in central Australia (*10*). All animals were seronegative by both Luminex and VNT before challenge. Antibody was first detected by Luminex on day 10 or day 12 in each animal (Table 2); neutralizing antibody titers were 1:20 to 1:40 in alpaca 2 from day 10. Neutralizing antibody titers of 1:10 to 1:20 were detected in alpaca 1 from day 21 on but not in alpaca 3 at any time during the study. For controls, we used MERS-CoV positive and negative serum samples from Egypt and Australia (Technical Appendix Table). 

**Table 2:** Serologic responses in 3 infected alpacas, as measured by virus neutralization tests and Luminex bead assays for selected sample days*

Dpi	Serologic results								No. positive/no. tested)§	
	VNT titer†				Luminex assay MFI‡					
	Alpaca 1	Alpaca 2	Alpaca 3		Alpaca 1	Alpaca 2	Alpaca 3		VNT	Luminex
0	Negative	Negative	Negative		787	889	167		0/3	0/3
3	Negative	Negative	Negative		373	814	152		0/3	0/3
5	Negative	Negative	Negative		418	945	223		0/3	0/3
7	Negative	Negative	Negative		272	932	249		0/3	0/3
10	Negative	**1:40**	Negative		478	**2,869**	58		1/3	1/3
12	Negative	**1:40**	Negative		928	**10,274**	331		1/3	1/3
14	Negative	**1:40**	Negative		1,041	**7,658**	899		1/3	1/3
21	1:10	**1:40**	Negative		877	**6,893**	629		1/3	1/3
24	**1:20**	**1:20**	Negative		1,506	**3,324**	678		2/3	1/3
26	**1:20**	**1:20**	Negative		853	**4,161**	667		2/3	1/3
28	**1:20**	**1:20**	Negative		773	**4,682**	724		2/3	1/3
31	**1:20**	**1:40**	Negative		548	**11,259**	649		2/3	1/3
33	1:10	**1:20**	Negative		688	**6,090**	455		1/3	1/3
35§	1:10	NA	Negative		510	NA	586		0/2	0/2

*Bold indicates positive results. Gray shading indicates that >1 animal was positive for the sample collected on that day. Dpi, day postinfection for challenge (initial infection); MFI, median fluorescent intensity; VNT, virus neutralization test. †The starting dilution was 1:10, and the threshold for a positive result was a dilution of >1:20.  ‡The MFI threshold for a positive result for the Luminex assay was 2,500.  §Alpaca 2 was euthanized at day 33, leaving only 2 animals in the study at day 35.

## Conclusions

Our study confirms that alpacas are susceptible to MERS-CoV infection; this finding is consistent with a previous report showing that alpaca kidney cell lines possessing the dipeptidyl peptidase-4 receptor could be infected in vitro (*11*). Our challenge/rechallenge trial was planned as a first stage in the assessment of the alpaca as a potential surrogate for camels for MERS-CoV vaccine testing. Consequently, the trial was not designed for direct comparison with 2 previous MERS-CoV challenge trials reported in camels (*5**,**6*). Our trial used a lower challenge dose and a different timeframe for observation; nevertheless, some preliminary comparative observations may be useful. In the previous studies, as in ours, the animals were inoculated by the oronasal route, and live virus was detected through day 7 postinfection. Similarly, neutralizing antibodies were detected beginning 7–8 days postinfection. However, findings in the trials with camels differed considerably from findings in our trial. The trials with camels detected live virus from nasal washes at days 1–3, a nasal discharge, and transient temperature rises; viral RNA was detected by real-time PCR for an extended period. Furthermore, the VNT titers for camels were much higher than those for the alpacas in our study. These differences possibly represent underlying dissimilarities in immune responses to MERS-CoV for the 2 species but may also result from the higher infecting dose (10^7^ 50% tissue culture infective dose) used in the camel studies.

Our study showed that alpacas secreted live virus after oronasal infection and that the immune response to the initial infection prevented further excretion following reinfection. An underlying assumption in our trial is that the initial infection equates to natural vaccination and that the lack of viral excretion thus follows an induced immune memory response. However, our results indicate that this immunologic response is complex; although a strong serologic response developed in only 1 alpaca, all 3 alpacas were refractory to reinfection.

This study has several limitations. First, it was a preliminary study with only 3 animals and functioned more as proof of concept than a definitive study of the use of alpacas as a model for studying infection dynamics of MERS-CoV in camelids. Second, our observation period of 21 days before rechallenge is informative but does not provide complete information on duration of protective immunity. Future studies should have a larger sample and a longer period of study postinoculation. Third, our study did not seek to understand the pathogenesis of infection; we did not conduct histopathology or immunohistochemistry to understand the site of initial viral replication and the role of mucosal immunity in mounting an effective immune response upon infection.

Notwithstanding these limitations, we believe that the alpaca might be a useful model that could greatly facilitate the development and testing of vaccine candidates. We recommend further research and trials to substantiate this potential.

**Note Added in Proof:** Adney et al. also report infection, replication, and transmission of Middle East respiratory syndrome coronavirus in alpacas in this issue of Emerging Infectious Diseases (*12*).

**Technical Appendix.** Results from virus neutralizing tests and Luminex assays for control sera used in study of 3 alpacas experimentally infected with MERS-CoV.
