# Supplementary material for: Experimental Infection and Response to Rechallenge of Alpacas with Middle East Respiratory Syndrome Coronavirus
Source: Emerg Infect Dis. 2016 Jun;22(6):1071–4. doi: 10.3201/eid2206.160007 (PMC4880109; doi:10.3201/eid2206.160007)
Supplement: Supplementary file 1 — Technical Appendix. Results from virus neutralizing tests and Luminex assays for control sera used in study of 3 alpacas experimentally infected with MERS-CoV. [file 16-0007-Techapp-s1.pdf]

Article DOI: http://dx.doi.org/10.3201/eid2206.160007

Experimental Infection and Response to Rechallenge of Alpacas with Middle East Respiratory Syndrome Coronavirus

Technical Appendix Table. Results from virus neutralizing tests and Luminex assays for control sera used in study of rechallenge of alpacas with Middle East respiratory syndrome coronavirus*

Control type	Country of origin	VNT titre†	Luminex assay (MFI)
Positive 1	Egypt	>1:1,280	10,746
Positive 2	Egypt	>1:1,280	20,599
Positive 3	Egypt	1:640	4,689
Negative 1	Egypt	Negative	85
Negative 2	Australia	Negative	691
Negative 3	Australia	Negative	845
Negative 4	Australia	Negative	491
Negative 5	Australia	Negative	303

*MFI, median fluorescent intensity; VNT, virus neutralization test.
†Starting dilution was 1:10. Positive results are those showing virus neutralization at a dilution of ≥1:20.
